# A dual mechanism underlying retroactive shifts of auditory spatial attention: dissociating target- and distractor-related modulations of alpha lateralization

**DOI:** 10.1038/s41598-020-70004-2

**Published:** 2020-08-17

**Authors:** Laura-Isabelle Klatt, Stephan Getzmann, Alexandra Begau, Daniel Schneider

**Affiliations:** Leibniz Research Centre for Working Environment and Human Factors, Ardeystraße 67, 44139 Dortmund, Germany

**Keywords:** Working memory, Cognitive neuroscience, Attention, Neuroscience

## Abstract

Attention can be allocated to mental representations to select information from working memory. To date, it remains ambiguous whether such retroactive shifts of attention involve the inhibition of irrelevant information or the prioritization of relevant information. Investigating asymmetries in posterior alpha-band oscillations during an auditory retroactive cueing task, we aimed at differentiating those mechanisms. Participants were cued to attend two out of three sounds in an upcoming sound array. Importantly, the resulting working memory representation contained one laterally and one centrally presented item. A centrally presented retro-cue then indicated the lateral, the central, or both items as further relevant for the task (comparing the cued item(s) to a memory probe). Time–frequency analysis revealed opposing patterns of alpha lateralization depending on target eccentricity: A contralateral decrease in alpha power in *target lateral* trials indicated the involvement of target prioritization. A contralateral increase in alpha power when the central item remained relevant (*distractor lateral* trials) suggested the de-prioritization of irrelevant information. No lateralization was observed when both items remained relevant, supporting the notion that auditory alpha lateralization is restricted to situations in which spatial information is task-relevant. Altogether, the data demonstrate that retroactive attentional deployment involves excitatory and inhibitory control mechanisms.

## Introduction

In everyday life, we frequently rely on selective attention in order to focus on information that is relevant while ignoring behaviorally irrelevant sensory input. Without such an attentional filter, we would be overwhelmed by the sheer abundance of sensory information. Analogously, selective attention can operate on working memory contents that are no longer physically present in the environment. Such retroactive shifts of attention are critical in order to adapt to changing task demands and allow for an efficient allocation of limited mental storage resources. The deployment of covert spatial attention to one side in mnemonic (or perceptual) space has been linked to spatially-specific modulations of alpha oscillations^1–3^. Typically, there is a relative decrease in alpha power over posterior scalp sites contralateral to the attended location, while alpha power increases contralateral to the unattended location. Based on the gating-by-inhibition framework by Jensen and Mazaheri^4^, low alpha power has been proposed to reflect a state of high excitability in the respective neural areas, whereas high alpha power reflects the functional inhibition of task-irrelevant regions.

Analogously, two mechanisms could underlie the selection of information from working memory: shifting attention within working memory may either *facilitate* or *strengthen* the relevant information, or, on the other hand, the no longer relevant contents may be *inhibited* and thereby dropped from the focus of attention within working memory. Although many studies investigating alpha lateralization interpret their findings in terms of an inhibition account, very few have been successful in actually dissociating those two mechanisms^5^. That is largely due to the fact that the majority of studies has used lateralized stimulus displays, in which targets and distractors are presented in opposite hemifields. Thus, a lateralization of alpha power in response to a shift of attention towards a left-sided target can be likewise due to a contralateral (i.e., right-hemispheric) decrease in alpha power (reflecting target prioritization) or to an ipsilateral (i.e., left-hemispheric) increase in alpha power (reflecting distractor inhibition). Notably, the same debate and issues apply to the perceptual domain, that is, to the focusing of attention on relevant aspects of the external environment (for a review, see^6^).

Here, we aimed at distinguishing those mechanisms using an auditory working memory paradigm. In an auditory retroactive cueing task (design adapted from a previous experiment in the visual modality: Schneider et al.^7^), participants were initially cued to attend two out of three sounds in an upcoming sound array. In any case, the resulting working memory representation contained one laterally (left or right) and one centrally presented item. A retroactive cue (retro-cue) then indicated either one (i.e., selective retro-cue) or both (i.e., neutral retro-cue) of those items as further relevant for the task, which required participants to compare the cued item(s) to a centrally presented probe stimulus. Participants were instructed to indicate whether the probe stimulus was equal to the retro-cued item(s) or not. The probe stimulus could be either a new sound (i.e., a sound that never appeared in the given trial; no response), the cued sound (i.e., the sound indicated as relevant by the retro-cue; yes response), or the non-cued sound (i.e., the sound indicated as irrelevant by the retro-cue; no response).

**Figure 1:**
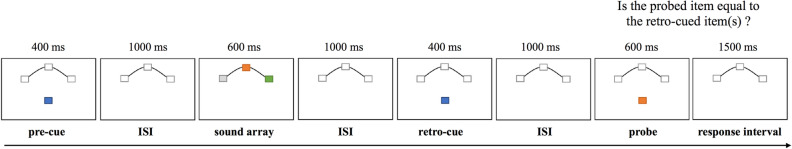
Schematic illustration of the task design. The three front loudspeakers were located at azimuthal positions of − 90°, 0°, 90° in the horizontal plane. The back loudspeaker, through which pre-cue, retro-cue, and probe were presented, was located right behind the participant’s head at an approximate distance of 30 cm. *ISI* inter-stimulus-interval.

This design (Fig. 1) entails two major strengths that differ from previous (predominantly visual) retro-cueing studies. First, the retro-cue (as well as the pre-cue) was presented from a central position behind the participants’ head, eliminating the risk that associated EEG asymmetries reflect the processing of the cue rather than the attentional selection or inhibition of working memory items. Second, the key aspect of this design was that only either the target (i.e., the cued item) or the distractor (i.e., the non-cued item) was lateralized. This spatial arrangement of stimuli in the sound array was essential to make sure that hemispheric asymmetries in the alpha frequency-band following the retro-cue could be unambiguously linked to either the processing of the target or the distractor. The reasoning behind this design is based on the organization of afferent auditory connections and the resulting implications for hemispheric differences in processing: It is known that the contralateral projections, transmitting auditory input, are stronger and more preponderant than the ipsilateral projections^8^. Thus, the contralateral hemisphere should predominately process the attended stimulus, whereas the ipsilateral hemisphere should predominantly process the unattended stimulus. A centrally presented sound, however, should be equally represented in both hemispheres. According to this, if the selection of information from auditory working memory involves the spatially-specific inhibition of irrelevant information at a lateralized position, a contralateral increase in alpha power should be evident when the central working memory item remains relevant (i.e., when the lateral, non-cued item becomes irrelevant, *distractor lateral condition*). Here, the non-cued item is the only lateralized stimulus; hence, alpha lateralization should be unambiguously related to the processing (i.e., inhibition) of the distractor. If, however, retroactive shifts of auditory attention involve the prioritization of relevant information, a contralateral decrease in alpha power should be evident when the lateral working memory item remains relevant (i.e., when the central, non-cued item becomes irrelevant; *target lateral condition*). Again, since the cued item is the only lateralized stimulus in that case, any hemispheric asymmetry in the alpha frequency band can solely be related to the processing of the target. Obviously, those two possibilities are not mutually exclusive and may as well both contribute to successful selection of information. Yet, the experimental design allows us to distinguish the two from one another. A third neutral retro-cue condition, in which both items remained relevant, served as a control condition. A neutral cue did neither require a re-orienting of attention within working memory nor the access to the stored spatial position of the memorized sounds. Instead, once the neutral retro-cue appeared, it was sufficient to maintain the sounds’ identities. Thus, in line with previous results, showing that alpha lateralization is limited to situations in which spatial position is a task-relevant feature^9^, we did not expect an asymmetry following neutral retro-cues.

## Results

### Behavioral results

To investigate whether a selective retro-cue, allowing for working memory updating and a reduction in working memory load, led to an improvement in task performance compared to neutral retro-cues, we conducted paired sample *t*-tests for response times and accuracy. Participants performed significantly faster (*t*_(19)_ = − 7.84, *p* < 0.001, *p*_adj_ < 0.001, *g* = − 0.65, BF > 1,000) when only one item remained relevant (i.e., selective retro-cue trials) than when both items remained relevant (i.e., neutral retro-cue trials). Accuracy did not differ between the two conditions (*t*_(19)_ = 0.72, *p* = 0.481, *p*_adj_ = 0.721, *g* = 0.09, BF = 0.29).

In addition, to differentiate the effects of different probe types (cued, non-cued, new), we performed a one-way repeated-measures analysis of variance (rANOVA) for response times and a Friedman’s ANOVA for accuracy. This analysis allows us to shed light on the fate of the non-cued information; that is, it demonstrates whether or not the non-cued item (which was indicated as *irrelevant* by the retro-cue) was completely removed (i.e., successfully inhibited) from working memory. Critically, although non-cued items could be probed, the retro-cue was always 100% valid (i.e., knowing the cued item was always sufficient for solving the task). That is, there was no incentive for participants to retain the non-cued item in working memory. We expected selective retro-cue trials probing the non-cued item to result in slower and less accurate responses compared to trials in which the probe had never appeared in the current trial (i.e., new probe). That is, due to the interference from previously relevant information (i.e., the non-cued item), performance was expected to decline. For this comparison to work properly, neutral trials were excluded from the rANOVA, since there was no “non-cued item” when both items remained relevant.

**Figure 2:**
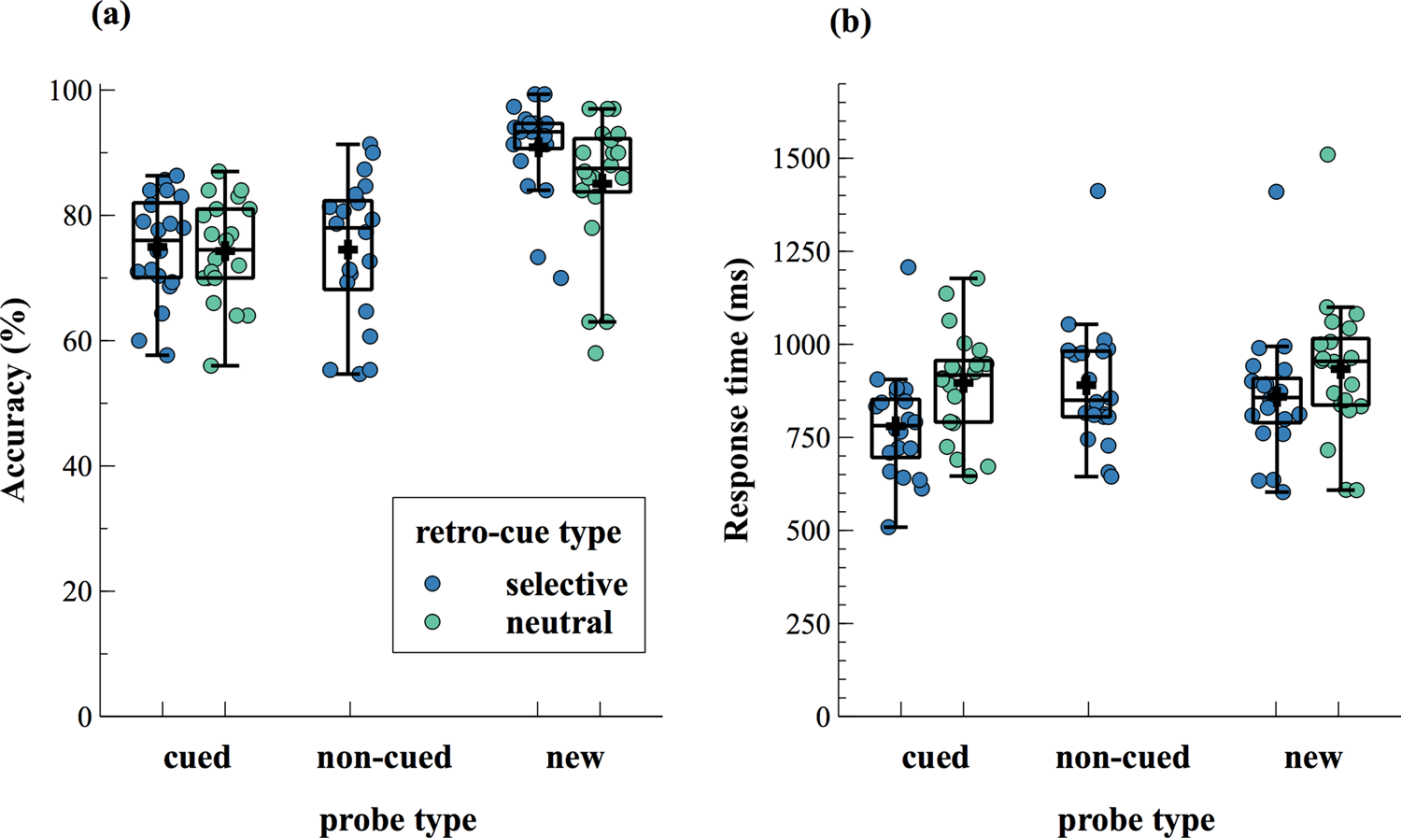
Accuracy (**a**) and response times (**b**), depending on retro-cue type and probe type. Boxplots illustrate the interquartile range and the median. Whiskers extend to 1.5 times the interquartile range. The dots indicate individual participants’ mean scores per condition. A black cross denotes the mean values across subjects for each condition. Note that in neutral retro-cue trials both items remained relevant and thus, a “cued probe” could be either one of those two sounds.

**Figure 3:**
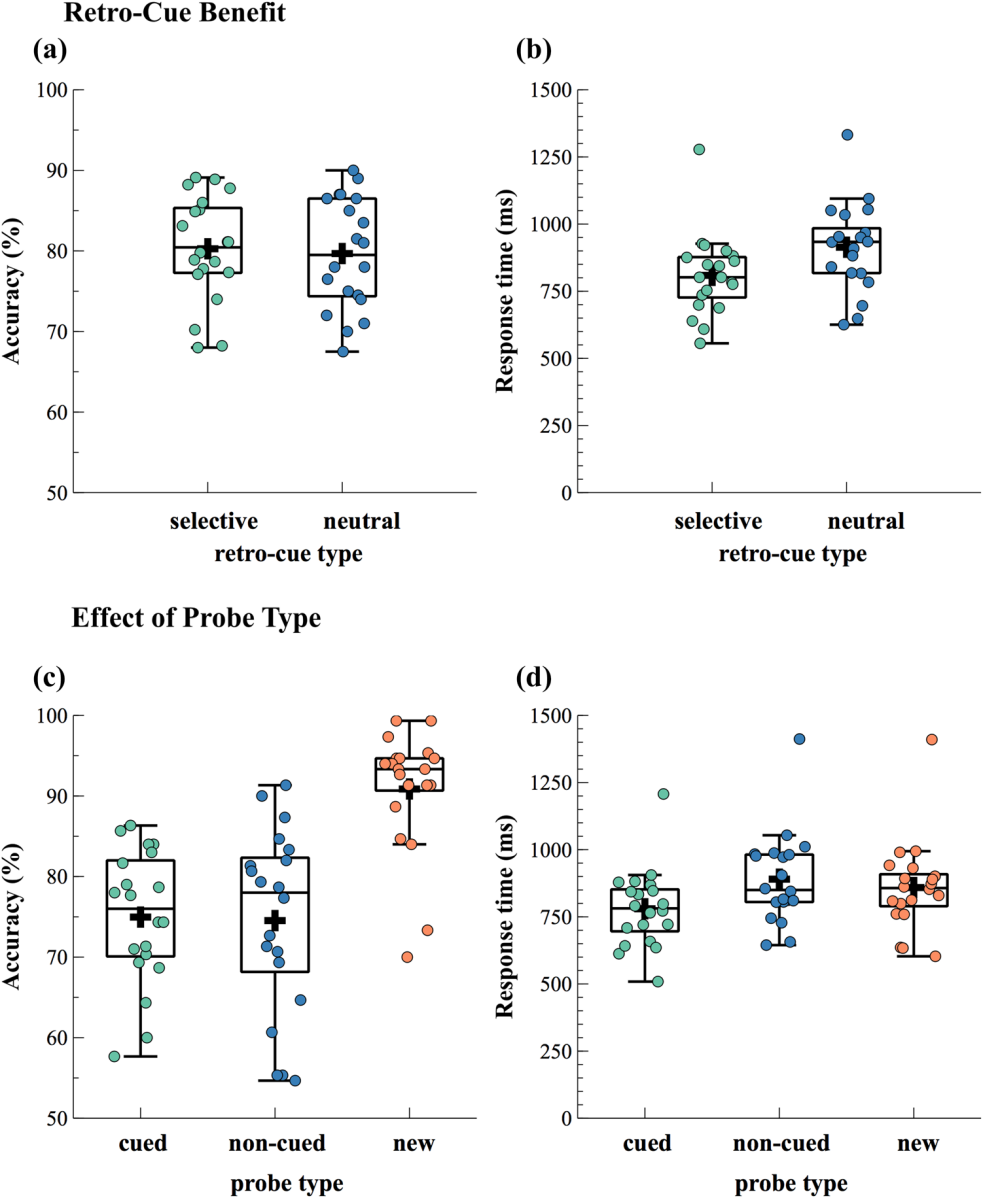
Accuracy (**a**,**c**) and response times (**b**,**d**), depending on the comparisons considered for statistical analyses. To test for benefits of selective retro-cues, performance in selective and neutral retro-cues was compared, using paired sample *t*-tests. Because in neutral retro-cue trials, it was not possible to probe a non-cued item, the data depicted for selective retro-cues do not include non-cued probe trials (**a**,**b**). Accuracy (**c**) and response times (**d**), depending on probe type, refer exclusively to selective retro-cue trials because neutral retro-cue trials did not allow for the main comparison of interest between non-cued and new items. A repeated-measured ANOVA was conducted to test for effects of probe type. For further details on statistical analyses see the methods section. Boxplots illustrate the interquartile range and the median. Whiskers extend to 1.5 times the interquartile range. The dots indicate individual participants’ mean scores per condition. A black cross denotes the mean values across subjects for each condition.

The rANOVA revealed a significant effect of probe type on response times (*F*_(2,38)_ = 39.56, *p* < 0.001, *p*_adj_ < 0.001, ηp^2^ = 0.68, ε = 0.93) as well as on accuracy (χ^2^_(2)_ = 27.1, *p* < 0.001, *p*_adj_ < 0.001). In line with our hypotheses, post-hoc comparisons revealed that participants responded slower (*t*_(19)_ = 2.59, *p* = 0.018, *p*_adj_ = 0.033, *g* = 0.18, BF = 3.17) as well as less accurate (*t*_(19)_ = − 10.32, *p* < 0.001, *p*_adj_ < 0.001, *g* = − 1.63, BF > 1,000) in trials with non-cued compared to new probes. In addition, participants responded significantly faster (*t*_(19)_ = − 5.50, p < 0.001, *p*_adj_ < 0.001, *g* = − 0.49, BF = 917.85) as well as less accurate (*t*_(19)_ =  − 7.05, *p* < 0.001, *p*_adj_ < 0.001, *g* = − 1.95, BF > 1,000) when the cued item was probed as opposed to a new item. When contrasting trials with cued versus non-cued probes, post-hoc tests showed a significant difference in response times (*t *_(19)_ = − 9.38, *p* < 0.001, *p*_adj_ < 0.001, *g* = − 0.68, BF > 1,000), but not in accuracy (*t*_(19)_ = 0.17, *p* = 0.871, *p*_adj_ = 1.60, *g* = 0.04, BF = 0.24). The described behavioral results are illustrated in Figs. 2 and 3.

### Alpha lateralization as a correlate of target prioritization versus Inhibition

To differentiate whether attentional selection within auditory working memory is associated with target prioritization or distractor inhibition, we calculated the Alpha Lateralization Index (ALI) for target lateral, distractor lateral, as well as neutral retro-cue trials. The ALI indicates the ratio of ipsilateral minus contralateral alpha power and the total power across both hemispheres [ALI = ipsilateral − contralateral alpha power / ipsilateral + contralateral alpha power]. Ipsilateral and contralateral refer to the hemispheres (or electrode positions) relative to the lateralized item. Thus, positive ALI values indicate a contralateral decrease of alpha power (i.e., the processing of the lateralized information) and negative ALI values indicate a contralateral increase of alpha power (i.e., the suppression of the lateralized information).

**Figure 4:**
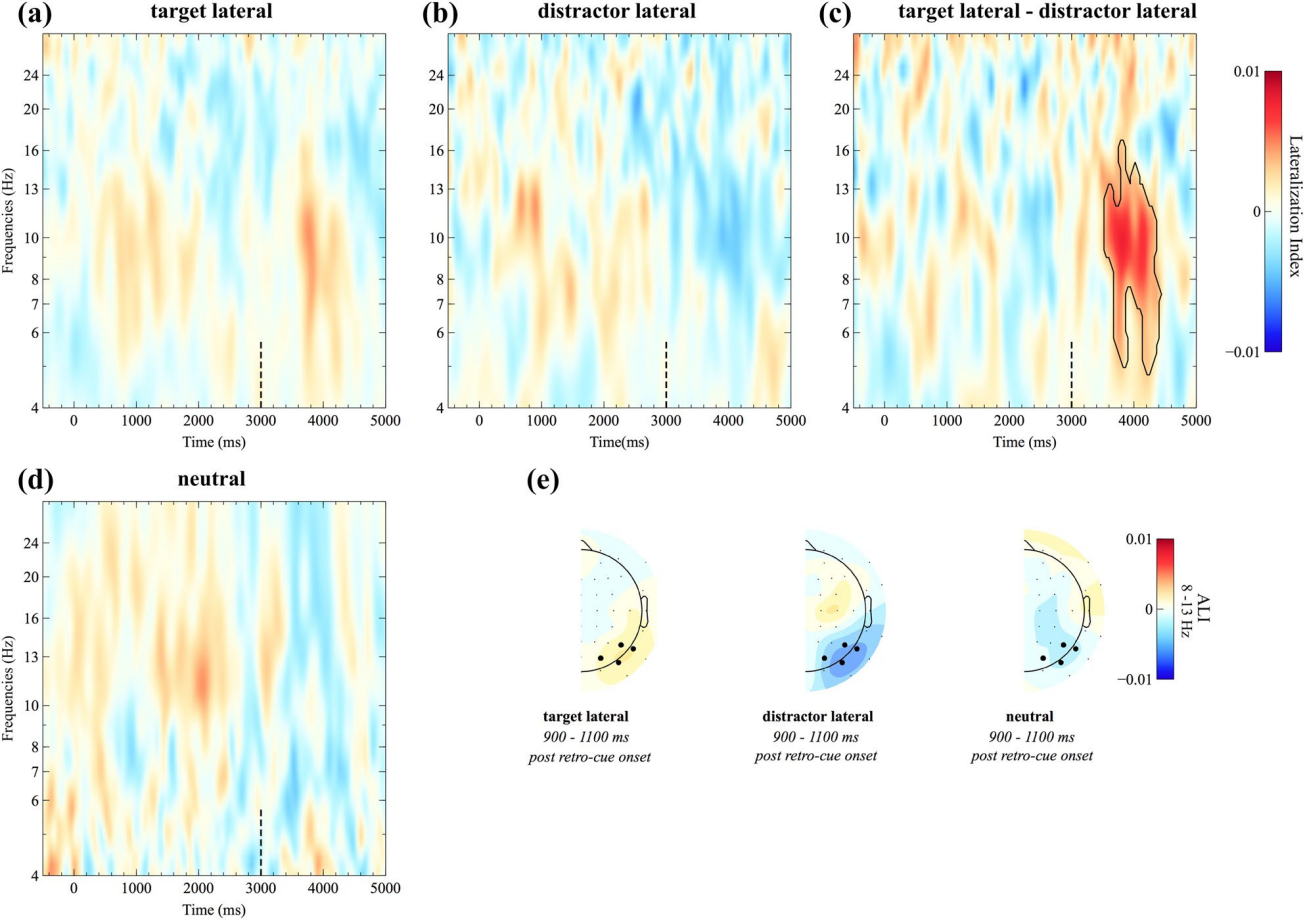
Alpha lateralization results for target lateral, distractor lateral, and neutral trials. Time–frequency plots (**a**,**b**,**d**) show the average lateralization indices at posterior electrodes (PO7/8, P7/8, P5/6, and PO3/4) per condition. The black dashed line indicates retro-cue onset. Target lateral and distractor lateral trials were contrasted using cluster permutation statistics. The resulting significant cluster (*p* < .05 and size > 95th percentile of the null distribution) is framed by a black line in the target lateral minus distractor lateral difference plot (**c**). Corresponding topographies are based on the normalized difference between ipsilateral and contralateral alpha power following the retro-cue (**e**).

**Figure 5:**
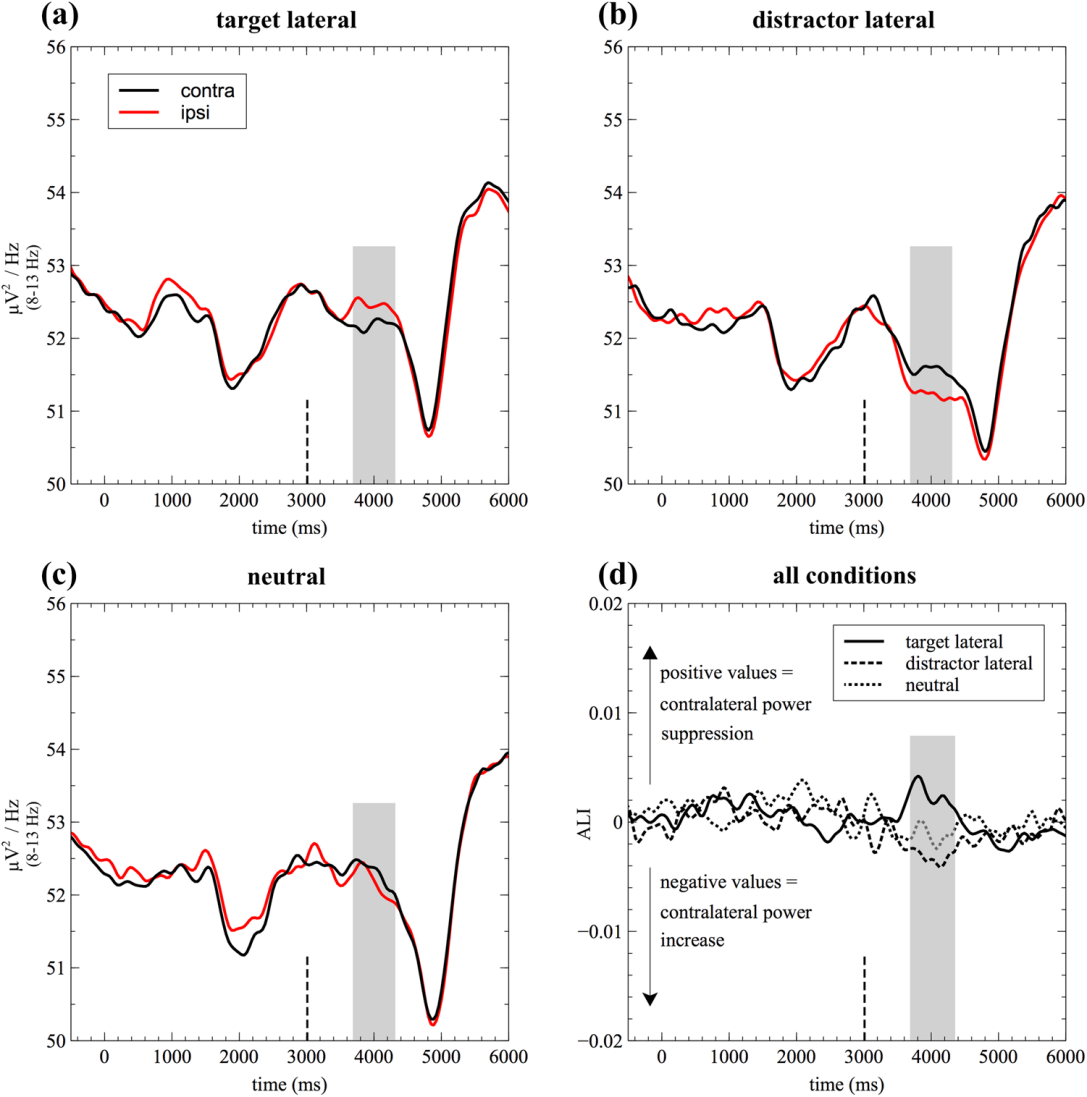
Alpha lateralization results depending on condition. Line plots for the target lateral (**a**), distractor lateral (**b**), and neutral condition (**c**) illustrate the contralateral and ipsilateral portion of alpha power (raw power values) for a posterior cluster of electrodes (PO7/8, P7/8, P5/6, and PO3/4). The bottom, right plot (**d**) shows the Alpha Lateralization Index (ALI) values for all three conditions. The analysis time window is highlighted in grey.

As illustrated in Figs. 4 and 5, opposite patterns of lateralization were observed when comparing target lateral (Figs. 4a, 5a) and distractor lateral trials (Figs. 4b, 5b). That is, target lateral trials resulted in a contralateral decrease of alpha power, whereas distractor lateral trials resulted in a contralateral increase of alpha power. A cluster-based permutation analysis confirmed this impression, revealing a significant difference between conditions in the alpha frequency band following the retro-cue (cluster size > 95th percentile of null-distribution and *p* < 0.05, Fig. 4c). Follow-up analyses were performed on mean alpha power (8–13 Hz) in an approximate time-window derived from this comparison, ranging from 700 to 1,300 ms post retro-cue onset: Paired-sample *t*-tests revealed a significant difference between neutral and target lateral trials (*t*_(19)_ = − 3.45, *p* = 0.003, *p*_adj_ = 0.016, *g* = − 0.98, BF = 15.46), whereas the difference between neutral trials (Figs. 4d, 5c) and distractor lateral trials failed to reach statistical significance (*t*_(19)_ = − 1.71, *p* = 0.104, *p*_adj_ = 0.297, *g* = − 0.52, BF = 0.80). Consistent with the reported *p *values, the BF provided strong support for the alternative hypothesis in the neutral versus target lateral comparison, whereas it was rather inconclusive for the neutral versus distractor lateral comparison.

In order to verify that the observed hemispheric asymmetries within conditions were significantly different from zero, one-sample *t*-tests were conducted: The results suggested that there was a reliable lateralization of alpha power in the target lateral (*t*_(19)_ = 2.85, *p* = 0.010, *p*_adj_ = 0.039, *g*_*1*_ = 0.64, BF = 5.07) as well as the distractor lateral condition (*t*_(19)_ = − 3.58, *p* = 0.002, *p*_adj_ = 0.016, *g*_*1*_ = − 0.80, BF = 20.20). With BFs greater than 3 and 10, respectively, those tests provide moderate and strong support for the alternative hypothesis (i.e., the lateralization is significantly different from zero). In contrast, there was no evidence for a lateralization of alpha power in the neutral retro-cue condition (*t*_(19)_ = − 1.54, *p* = 0.139, *p*_adj_ = 0.317, *g*_*1*_ = − 0.35, BF = 0.64). The corresponding BF did neither support the null nor the alternative hypothesis. Scalp topographies corresponding to the alpha lateralization indices are depicted in Fig. 4e. Related studies from the visual domain have previously raised concerns that posterior alpha power asymmetries might be confounded by lateral saccadic eye movement artifacts. See the supplementary material (Appendix A1) for a control analysis, invalidating such confounding effects.

In addition to alpha lateralization, the line plots in Fig. 5a–c clearly illustrate that there is a bilateral suppression of alpha power following retro-cue onset. For an analysis of non-lateralized alpha power modulations, we refer the reader to the supplementary material (Appendix A2).

## Discussion

The present study investigated the mechanisms underlying retroactive attentional orienting within auditory working memory. We demonstrated that signatures of target-related and distractor-related alpha lateralization can be dissociated. Specifically, by manipulating the spatial arrangement of targets and distractors such that only one of them appeared in a lateralized position, we were able to show that a retro-cue induced shift of attention towards a *lateralized target* resulted in a contralateral *decrease* in alpha power; in contrast, shifting attention to a centrally presented target and away from a *lateralized distractor* resulted in a contralateral *increase* in alpha power. The findings support the interpretation of alpha lateralization as a dual mechanism underlying the deployment of attention within working memory.

### Retro-cue benefits: effects on accuracy and response times

On the behavioral level, participants clearly benefited from selective retro-cues, reducing the amount of relevant information to be maintained in working memory. While participants were initially required to maintain two sound stimuli and their respective locations, following a selective retro-cue, only one item remained relevant. This resulted in a behavioral improvement in terms of faster response times compared to neutral retro-cue trials, in which both the lateral and the central item remained relevant. A large body of literature has investigated the mechanisms underlying such “*retro-cue benefits*” (for a review, see^10^), showing that focusing attention within working memory results in the *strengthening* of attended working memory representations^11,12^ and the *removal* of non-cued information from a state of active maintenance (i.e., through persistent neural activity) in working memory^3,13^, thereby releasing resources for further processing^14^. When looking specifically at selective retro-cue trials and the different types of probe items, we found that participants responded the fastest when the probe matched the current working memory content (i.e., the cued item, yes response). However, in comparison to new probe items (no response), this speed benefit for cued items was associated with higher error rates. Given that those two conditions required different types of responses (yes vs. no), this can be most likely attributed to a speed-accuracy trade off.

Critically, we found increased response times and decreased accuracy in non-cued compared to new probe trials, suggesting a proactive interference effect in non-cued probe trials. It should be noted that although non-cued items were probed in a subset of trials, the retro-cue was always 100% valid. That is, when participants were asked to judge whether the probe item matched the cued item, it made no difference whether the probe was a new item or the previously non-cued item. As long as they remembered the (validly) cued item, they would always be able to respond correctly. Hence, the question emerges, if the non-cued item is *removed* from working memory, why does it still cause interference when presented as a probe item? If the non-cued item had been completely removed from short-term storage, responses should not have differed from those to new probe items. Instead, the present results suggest that the non-cued item was still maintained outside the focus of attention. This is in line with a previous visual retro-cueing study, similarly showing an interference effect, that is, slower response times for non-cued compared to new probe items^3^. By contrasting the ERP response to non-cued and new probes, Schneider et al.^3^ demonstrated that an additional recollection process was involved in the rejection of the non-cued item. Critically, the ERP response to the probe also indicated that participants always focused their attention onto the cued item in all probe conditions, rather than shifting attention back towards the non-cued item, yet, the related contralateral negativity was more sustained in non-cued probe trials. The authors thus argue that while new probes can be easily rejected based on a fast familiarity process, non-cued probes require a more time-demanding comparison process through prolonged focusing on the cued item. Given the very comparable design of our auditory retro-cueing task, we assume similar processes might be involved in the present study.

The presence of a “residual” representation of non-cued information is also in line with retro-cue studies incorporating invalidly cued trials, which consistently suggest that non-cued items are not irreversibly lost. Those studies reliably report a decline in performance when the non-cued item was invalidly probed; however, performance never dropped to chance level^15,16^. In addition, double-cueing paradigms suggest that the information that was initially dropped from working memory (in response to a first cue) can be restored to some extent by a second retro-cue^11,17^. Such findings corroborate a recently proposed framework of “activity-silent” working memory^18^, suggesting that persistent neural firing is not a necessary prerequisite for successful short-term memory maintenance. For instance, using multivariate pattern analysis, it could be convincingly demonstrated that the active neural signature representing currently maintained information disappears once they become temporarily irrelevant. Critically, their neural signature is restored once those items are again cued to be relevant for the task^19,20^. Accordingly, these studies suggest that non-cued items are *removed from the focus of attention* and from a state of persistent neural activity, but are still available for reactivation by re-storing their neural signature (see^21,22^ for alternative accounts regarding the storage of un-prioritized items). However, the reactivation of information from activity-silent representations was originally only investigated for information that remained task-relevant (e.g., at a later point in time). In contrast, when a retro-cue indicated the to-be-probed item with 100% validity, Wolff and colleagues were not able to decode the irrelevant (non-cued) item from a hidden state in the EEG signature^23^. This contradicts the present findings as well as those previously reported by Schneider et al., both of which used perfectly valid retro-cues. Yet, Bae and Luck^24^ recently reported that the reactivation of task-irrelevant information from the previous trial in a working memory task was possible.

Taken together, we argue that in the present study, the non-cued information was removed from the focus of attention, but remained in a state of alternative, potentially passive storage, which caused a conflict in non-cued probe trials and thus, might have been reactivated by the current retrieval context (i.e., the probe)^19^. Due to this conflict, non-cued probe trials required a more time-consuming and more error-prone comparison process to correctly reject the probe item. After all, although the residual representation of the non-cued item clearly caused a performance decline (cf. intrusion costs in a modified Sternberg paradigm^25^), we need to acknowledge that we cannot rule out that participants strategically held on to the non-cued item in an altered form, simply because they figured it would sometimes appear as a probe stimulus. We will further discuss the underlying mechanisms of such “imperfect” removal below in the context of the obtained time–frequency results.

### Alpha lateralization as a proxy of target prioritization and distractor inhibition?

On a neural level, shifts of attention in response to the retro-cue were reflected by a hemispheric lateralization of posterior alpha lateralization. Critically, a contralateral decrease in alpha power was evident in target lateral trials, whereas a contralateral increase in alpha power was evident in distractor lateral trials. Broadly speaking, this is consistent with a growing body of literature showing that attentional modulations of alpha oscillations are comparable for shifts of attention within perceptual space and within working memory^2,9,26^. More specifically, this pattern of results replicates previous findings from an analogous visual retro-cueing study^7^, revealing dissociable modulations of target- and distractor-related alpha power when shifting attention within visual working memory representations.

Given that it is relatively undisputed that alpha lateralization tracks the attended location within working memory^27–29^, the observed spatially-specific modulations clearly indicate a re-orienting of spatial attention towards relevant items (and away from irrelevant items). Consequentially, the relevant item is moved into a prioritized state, commonly referred to as the focus of attention^30^, which renders the selected information more accessible for future cognitive operations. In line with that, because the cued information is already selected and ready to be acted on, cued probes result in faster response times compared to non-cued and new probes. Consistently, decreases in alpha power have been previously associated with increased neural firing^31^ and improved behavioral performance^31–33^, suggesting that the observed target-related alpha power decrease reflects a signature of target prioritization, that allows for a fast adaption to current task demands. More specifically, it has been hypothesized that such a prioritization may result from the attentional strengthening of relevant items to their (spatial) context^11^.

Whether in addition to a prioritization mechanism, (active) inhibition is also involved in the attentional selection from working memory remains a matter of debate. The majority of previous studies, using completely lateralized stimulus displays, do not allow for a clear distinction between an inhibition or a prioritization account^5^. Here, we demonstrated a relative distractor-related alpha power increase when participants were cued to re-orient their attention to a non-lateralized (i.e., central) working memory item, or—in other words—when shifting their attention away from a lateralized distractor. Increases in alpha power have been linked to decreased neuronal firing rates^31^ and have been shown to occur over task-irrelevant cortical areas^34^ as well as in anticipation of distractors^35^. Hence, interpreting the observed alpha power increase in terms of an inhibition mechanism appears plausible. Yet, the exact nature of such an inhibitory attentional signature still remains elusive. First of all, we need to acknowledge that we cannot be certain that the inhibitory process, as indicated by the distractor-related alpha modulations, is independent from the attentional template for the target. Thus, it remains possible that distractor inhibition is an automatic consequence of target prioritization. Further, if we do conceptualize distractor inhibition as a top-down control mechanism that deteriorates the respective representations^36^, the question brought forth above likewise applies: If non-cued items are inhibited, why do non-cued items still cause interference when presented as a probe stimulus? Accordingly, both behavioral as well as neural evidence question the notion that an (active) inhibition mechanism completely deteriorates the non-cued item: On a behavioral level, using a series of cues, it has been shown that originally cued and then defocused items remain strengthened and result in faster and more accurate performance compared to trials in which a previously un-cued item (and thus “un-strengthened” item) is probed^11^. In addition, a number of EEG studies has cast doubt on whether alpha lateralization actually mediates potential distractor exclusion effects^37,38^: For instance, Noonan and colleagues failed to find a lateralization of alpha power in response to a pre-cue indicating the location of an upcoming distractor, although, on the behavioral level, clear response time and accuracy improvements provide evidence for effective distractor exclusion (see also de Vries et al.^39^). However, it should be considered that the latter findings refer to anticipatory suppression of distractors in the visual domain, whereas the present study deals with the inhibition of previously relevant information within auditory working memory that subsequently becomes irrelevant.

Taken together, it remains possible that alpha enhancement contralateral to the distractor may be related to the temporal suppression of maintenance of information in a state of persistent activity (i.e., within the focus of attention), which does not necessarily alter the quality of unattended working memory contents^19,40^. Alternatively, we could conceptualize distractor inhibition as a mechanism that ultimately aims to permanently remove irrelevant items from working memory. Such a permanent removal has been conceptualized to operate through an un-binding mechanism, that unties the present item-context bindings^41^, rather than affecting the representation of the item itself. This is in line with recent evidence, demonstrating that alpha oscillations carry information about the maintained location (e.g., the “context” in the present case) but *not* about location-unrelated stimulus-features (e.g., the sound’s identity)^27^. Given that the present behavioral results argue against a complete removal of the un-cued item, such a mechanism would be expected to unfold gradually, resulting in weakened but not entirely removed representations or item-context bindings.

### A dual mechanism underlying alpha lateralization

Overall, although we can only speculate on the exact nature of an inhibitory attentional control mechanism within auditory working memory, it is clear that we can dissociate distinct target- and distractor-related modulations of posterior alpha power. This supports the notion that working memory is a highly-flexible system that allows for the dynamic adaption to changing task demands by means of a dual-control mechanism. Alpha oscillations may serve as the underlying substrate that allows for flexible control over the state of current working memory representations, switching between prioritized and deprioritized representations depending on their current relevance for the task^38^.

That the flexible prioritization and de-prioritization of working memory representation may, in fact, be mediated by two independent mechanisms, has been supported by a number of recent studies: Schneider et al.^7^ independently manipulated the meaning of a retro-cue such that it either indicated the to-be-remembered (remember-cue) or the to-be-forgotten (forget-cue) item(s). Their results indicated that both types of retro-cues elicited a target-related decrease in alpha power as well as a distractor-related increase in alpha power. Interestingly, the “cued process” (i.e., inhibition in forget-cue trials and target prioritization in remember-cue trials) always emerged first and was only later followed by its complementary counterpart (see also Poch et al.^42^). In addition to those studies revealing latency effects, Wöstmann and colleagues^43^ were able to demonstrate that alpha lateralization for target selection and distractor suppression were both different in strength and source origin as well as statistically uncorrelated. Evidence for the assumption that the strengthening of target representations can in fact occur independently from the de-prioritization of irrelevant information also comes from behavioral findings, illustrating how strategic considerations may affect the underlying control mechanisms^44^: When a retro-cue indicated the to-be-probed item with low validity (i.e. valid cue in 50% of trials), invalid-cue costs were largely absent, while participants still showed a clear benefit for validly cued trials. In line with the strengthening hypothesis, such a pattern of results suggests that it is, in fact, possible to prioritize or strengthen the relevant information without affecting the non-cued, irrelevant information. However, under conditions of high cue validity (i.e., valid cue in 80% of trials), high invalid-cueing costs were observed, suggesting that in addition to a target prioritization mechanism, non-cued items might have been “removed” in order to free working memory resources. Interestingly, in a separate study, Günseli et al. found that cue validity (50% vs. 80%) only affected contralateral, but not ipsilateral alpha power (relative to the cued item)^45^, which corroborates the above-mentioned claim that the decision to remove or inhibit an item from working memory appears to be separate from the decision to prioritize the relevant information. Contrasting this finding to the present results, pointing to both a target- and a distractor-related modulation of alpha power, one may speculate that the incentive to (actively) inhibit the irrelevant information increases with unambiguous, perfectly reliable retro-cues.

Finally, considering the broad consensus that there is a large overlap between attentional selection in the perceptual environment (i.e., external attention) and attentional selection within working memory (i.e., internal attention; see^46–48^ for elaborate reviews considering both domains), the question emerges to what extent the mechanisms examined in the present study may be comparable to respective processes in the perceptual domain. Similar to the working memory literature, facilitative effects of attention are well-characterized and widely accepted in perception, whereas it remains a matter of debate to what extent inhibition acts as an active, top-down controlled mechanism that is independent from the former (reviewed by van Moorselaar et al.^6^). In fact, a number of studies have demonstrated that shifts of external^49,50^ and internal attention^2,9,26^ yield roughly comparable signatures of alpha-band modulations, suggesting the reliance on a common underlying control mechanism. Surprisingly, despite the common notion that alpha oscillations reflect functional inhibition (see e.g., the gating by inhibition framework by Jensen and Mazaheri^51^), the empirical evidence associating anticipatory modulations of alpha oscillations with top-down inhibition still remains scarce (reviewed by^5,6^).

### Alpha lateralization is absent when spatial information becomes irrelevant

We did not observe any lateralization of alpha power following a neutral retro-cue, in which case both items maintained in working memory remained relevant. This appears plausible, considering that the neutral retro-cue did not require a re-orienting of spatial attention. In addition, this is consistent with the notion that auditory alpha lateralization is limited to situations in which spatial information is task-relevant^9,50^. Accordingly, once the neutral retro-cue appeared, the spatial location of the sounds became irrelevant to solving the task, as it was sufficient to know the sounds’ identities. In turn, this also means that alpha lateralization in response to a selective retro-cue reflects the spatially-specific access to the contents of working memory. This further corroborates the above-mentioned claim that alpha suppression indicates the strengthening of item-context bindings in working memory. In the present case, spatial location represents the item’s context, which is required to successfully distinguish cued from non-cued items. In contrast, in neutral retro-cue trials, knowledge of the spatial context is not required to solve the task.

Finally, the absence of alpha lateralization in neutral retro-cue trials points toward a critical difference between the auditory and the visual modality. Notably, Schneider et al.^52^, using a related design, found significant alpha lateralization following neutral retro-cues. This can be most likely attributed to the fact that the organization of the visual system is fundamentally different in that it is inherently spatial. Consistently, it has been shown that spatial locations are actively coded and maintained by population-level alpha-band activity throughout a non-spatial visual working memory task^29^.

## Conclusion

Taken together, using an auditory retro-active cueing design, we demonstrate that it is possible to unambiguously dissociate target- and distractor related modulations of alpha power oscillations. The results indicate that both excitatory and inhibitory attentional control mechanisms contribute to the selection of information from working memory. Accordingly, shifts of attention toward a lateralized target resulted in a contralateral decrease in alpha power, whereas shifting attention away from a lateralized distractor resulted in a contralateral increase in alpha power. The pattern of results support the notion that alpha lateralization mediates the task-dependent prioritization and de-prioritization of items within working memory^38^. In addition, we strengthen the previous claim that auditory alpha lateralization is absent when spatial information is task-irrelevant^9,50^.

## Methods

### Participants

Twenty-eight volunteers were paid to participate in this study. Four participants had to be excluded due to technical problems with their EEG recordings. Four additional participants showed excessive eye-movement artefacts (containing excessive lateral eye movements on more than 1/3rd of all trials) and were therefore excluded from further analyses. The remaining twenty participants (12 female) were aged between 19 and 28 years (mean age: 23.4 years) and right-handed as assessed using the Edinburgh Handedness Inventory^53^. All participants reported normal or normal-to corrected vision, no history of or current neurological or psychiatric disorders. Hearing acuity was assessed using an audiometry, including eleven pure-tone frequencies (0.125–8 kHz; Oscilla USB 100, Inmedico, Lystrup, Denmark). Hearing thresholds indicated normal hearing (≤ 25 dB hearing level). The study was conducted in accordance with the Declaration of Helsinki and approved by the Ethical Committee of the Leibniz Research Centre for Working Environment and Human Factors. Written informed consent was given by all participants prior to the beginning of the experimental procedure.

### Experimental setup and stimuli

The experiment took place in a dimly lit, echo-attenuated, sound-proof room. Participants were seated in front of a semi-circular array of eight broad-band loudspeakers (SC5.9; Visaton, Haan, Germany; housing volume 340 cm^3^) mounted in the horizontal plane. Three of those loudspeakers, located at azimuthal positions of − 90°, 0°, 90°, were used for sound presentation in the present study. One additional loudspeaker was located right behind the participant’s head at a distance of approximately 30 cm. A red light-emitting diode, attached below the central loudspeaker (diameter 3 mm, turned off) served as a central fixation point. The participants’ head was kept at a constant position using a chin rest.

Eight familiar animal sounds, chosen from an online sound archive^54^, served as experimental stimuli. The original sounds (‘birds chirping’, ‘dog barking’, frog croaking’, ‘sheep baaing’, ‘cat meowing’, ‘duck quacking’, ‘cow mooing’, ‘rooster crowing’) were cut to a constant duration of 600 ms (10 ms on/off ramp), while leaving the spectro-temporal characteristics unchanged. In addition, syllable sounds served as cue-stimuli, indicating a certain subset of sound positions as relevant in a given trial. That is, the first two or three letters of the German words for right (i.e. “rechts”), left (i.e. “links”), middle (i.e. “mitte”), and both (i.e. “beide”) were used to construct the six cue-stimuli (“*li*”, “*re*”, “*mi*”, “bei”, “*mi-li*”, “*mi-re*”). The words were spoken by a female speaker (mean pitch 199 Hz). All cues had a duration of 400 ms. The overall sound pressure level of the sound arrays (presented at frontal loud speakers) was about 65 dB(A), whereas single animal sounds and the speech cue-stimuli (presented from behind participants’ heads) were presented at a sound pressure level of 60 dB(A).

### Procedure and task

The present experiment is a modified and simplified version of a retroactive cueing paradigm previously used in an investigation of visual retroactive attentional orienting^7^. Each trial comprised a sequence of four acoustic stimulus events, consisting of pre-cue, sound array, retro-cue, and probe. A trial started with the presentation of a pre-cue (400 ms), instructing participants to attend either the central and the left loudspeaker (“*mi-li*”) or the central and the right loudspeaker (“*mi-re*”). Thus, in the subsequently upcoming sound array, containing three animal vocalizations, one lateral sound was always known to be completely irrelevant for the rest of the trial. The sound array appeared 1,000 ms after the pre-cue and was presented for 600 ms. Rather than just presenting two sounds to begin with, we incorporated a pre-cue and a three-sound array to warrant that both hemispheres were equally activated during the sensory processing of the sound array. While in the visual domain, this can be easily implemented by displaying an irrelevant grey bar (cf. the original design by Schneider et al.^7^), it required an additional pre-cue in the present study. Following a short delay (1,000 ms), a retroactive cue (400 ms) indicated either one (i.e., selective retro-cue) or both (i.e., neutral retro-cue; “*bei*”) of the items currently maintained in working memory as further relevant for the task. Selective retro-cue trials can be further subdivided into target lateral trials (i.e., the retro-cue indicated the lateral item as further relevant, “*li*” or “*re*”) or distractor lateral trials (i.e., the retro-cue indicated the central item as relevant whereas the lateral item becomes irrelevant, “*mi*”). Each of the four retro-cue types was presented equally often (25% of trials), to enforce that none of the sound positions were favored irrespective of cue-type. That resulted in a higher number of trials for target lateral retro-cues (“li” and “re”; 50% of trials) as opposed to distractor lateral (“mi”, 25% of trials) and neutral (“bei”, 25% of trials) retro-cues. Finally, 1,000 ms subsequent to the retro-cue, a probe stimulus was presented. The probe stimulus was either the item that became irrelevant following the retro-cue (“non-cued probe”), the retro-cued item (“cued probe”), or a sound stimulus that never appeared in a given trial before (“new probe). Note that following a neutral retro-cue, the “cued probe” could be either one of the two still relevant items. The animal vocalization which was initially presented at the third, irrelevant position in the sound array (as indicated by the pre-cue) never appeared as a probe to ensure that participants would always ignore it. Because non-cued and new probes required a NO response they constituted 25% of all trials, respectively, whereas cued probes, requiring a YES response, constituted 50% of all trials. Participants were instructed to indicate whether the probe item matched (one of) the retro-cued item(s) by giving a YES (50% of trials) vs. NO (50% of trials) response. Participants responded by pressing one out of two vertically aligned keys, using the index finger and thumb of their right hand. The assignment of response alternatives (yes vs. no) to keys was counterbalanced across participants. Responses had to be given within 1,500 ms following probe offset. All probe stimuli, as well as pre- and retro-cues, were presented from a loudspeaker located in the median plane behind the participant’s head. The experiment consistent of a total of 30 practice trials and 800 experimental trials. The latter were divided into eight task blocks of 100 trials each. In-between blocks, short self-paced breaks served the prevention of fatigue in the course of the experiment. All participants were presented with the same, pseudo-random sequence of trials.

### Data analysis

#### Behavioural data

Response times and percentage of correct responses served as measures of behavioural performance. Trials with responses that occurred after the pre-defined response period (i.e., within the inter-trial interval), pre-mature (i.e., < 200 ms) and missing responses (i.e., no button press) were considered as erroneous responses. For analysis of response times, only correctly answered trials were included. In order to verify that the paradigm resulted in a retro-cue effect^15^, we performed paired sample *t*-tests, contrasting selective versus neutral retro-cues, for response times and accuracy data, respectively. Note, that for this comparison the non-cued probe trials (which were not present for neutral retro-cues) were excluded to avoid an imbalance between conditions. In addition, to test for the hypothesized interference effect in non-cued probe types, we contrasted the different probe types within the selective retro-cue condition by means of a repeated-measures analysis of variance (rANOVA) for response times and a Friedman’s ANOVA for accuracy, both of which included the factor *probe type*. Since for both the response time data as well as the accuracy data, we conducted a total of two analyses on the same data set (i.e., test for retro-cue type as well as for probe type), *p*-values were FDR-corrected for multiple comparisons across those two sets of analyses. In addition, post-hoc paired-sample *t*-tests (or their non-parametric alternative, i.e., Wilcoxon signed-rank test) were conducted and corrected for multiple comparisons. Note that adjusted *p*-values can be greater than 1.

#### EEG recording and processing

The EEG was recorded with a sampling rate of 1,000 Hz from 64 Ag/AgCl passive electrodes (Easycap GmbH, Herrsching, Germany), using a QuickAmp-72 amplifier (Brain products, Gilching, Germany). The electrodes were arranged across the scalp according to the extended 10/20 scalp configuration. AFz served as the ground electrode. The average of all channels constituted the online-reference. Impedances were kept below 10 kΩ during recording. Further pre-processing of the data was run in MATLAB (R2018b) and EEGLAB^55^. First, a Hamming windowed sinc FIR high-pass (0.5 Hz, 0.25 Hz cut-off frequency, 0.5 Hz transition band width, filter order: 6,600) and low-pass filter (30 Hz, 30.25 Hz cut-off frequency, 0.5 Hz transition band width, filter order 440) were applied. Then, channels with a normalized kurtosis (20% trimming before normalization) exceeding 5 standard deviations of the mean were rejected, using the automated channel rejection procedure implemented in EEGLAB (M = 3.25 channels, range = 1–5). Anterior lateral channels (Fp1/2, AF7/8, AF3/4, F9/10) were excluded from channel rejection to ensure reliable identification of eye movements. Data were re-referenced to the average of all non-rejected channels. Epochs ranging from − 1,000 to 7,500 ms relative to the onset of the pre-cue were generated. A rank-reduced independent component analysis (ICA) was run on a subset of the data, down-sampled to 200 Hz and including every second epoch. To detect and remove independent components (ICs) reflecting eye blinks, vertical eye movements, and generic discontinuities, the EEGLAB plugin ADJUST^56^ was applied. In addition, for each IC, a single-equivalent current dipole model was estimated by means of a spherical four-shell head model^57^. Any components with a dipole solution exceeding a threshold of 40% residual variance were also removed. Taken together, on average 20.75 ICs (range = 7–33) were rejected. This was followed by an automatic trial rejection procedure, detecting and removing data epochs, containing data values exceeding a threshold of 5 standard deviations in an iterative procedure (threshold limit: 1000 μV, maximum % of trials rejected per iteration: 5%). On average, 163.3 (20.41%) trials (range 0–309) were rejected in the course of this procedure. Finally, data from channels that were originally rejected were replaced using spherical interpolation. For faster processing of time–frequency analyses (cf. below), the data were down-sampled to 500 Hz.

#### Time–frequency analysis

In order to extract spectral power, the epoched EEG data was convoluted with a complex Morlet wavelet. The frequencies of the wavelets ranged from 4 to 30 Hz, increasing logarithmically in 52 steps. Convolution began with a 3-cycle wavelet for the lowest frequency and increased linearly as a function of frequency with a factor of 0.5, resulting in a 11.25-cycle wavelet for the highest frequency. No spectral baseline-correction was applied, since the calculation of alpha lateralization indices requires raw power input. The resulting event-related spectral perturbation (ERSP) epochs ranged from − 582 to 7,080 ms relative to pre-cue onset. Lateralized effects in posterior alpha band power (8–13 Hz) in response to the retro-cue were assessed as a measure of retroactive attentional orienting. As a robust measure of lateralization, a lateralization index^58^ was calculated as follows:

$$ lateralization\; index = \frac{ipsilateral \;power - contralateral \;power}{{ipsilateral + contralateral \;power}} $$

Mean power across trials was extracted for each subject ipsilateral and contralateral relative to the lateralized item in a given trial (i.e. the target in target lateral and neutral trials or the distractor in distractor lateral trials). Based on the electrode cluster used by Schneider et al.^7^, whose design this study was based on, power was averaged across the following electrodes of interest: PO7/8, P7/8, P5/6, and PO3/4. Because we had a clear hypothesis regarding an expected difference between the target lateral and the distractor lateral conditions, we first contrasted those two conditions using a cluster-based permutation approach: In a first step, the original data from the two conditions were contrasted by means of paired *t*-tests, comparing the lateralization index at each time-point (i.e., 200) and each frequency (i.e., 52). Note that no space dimension exists here, because power was already averaged across the electrodes mentioned above. This resulted in a time by frequency matrix of *p*-values. In a second step, the condition labels (i.e., target lateral vs. distractor lateral) were randomly assigned to the actual data points and again, a paired *t*-test was run for each time–frequency point. This procedure was iterated 1,000 times and thus, resulted in a 52 × 200 × 1,000 matrix of *p*-values. For each iteration, the size of the largest cluster of time–frequency points with a *p*-value below 0.05 was determined, resulting in a distribution of maximum cluster sizes to be expected under the null hypothesis. The 95th percentile of this distribution served as a cut-off value against which the clusters from the true data were compared. That is, only clusters of time–frequency points with a *p*-value below 0.05 that were larger than the 95th percentile of the permutation-based distribution of maximum cluster sizes were considered significant.

Based on the approximate cluster time-window in the alpha frequency range (8–13 Hz) derived from this comparison (700 to 1,300 ms post retro-cue onset), we compared the neutral retro-cue condition to the target lateral as well as the distractor lateral condition by means of paired-sample *t*-tests. Note that time-windows determined based on a cluster-permutation approach should not be interpreted in terms of an exact on- or offset of an effect^59^. In addition, we tested for significant alpha lateralization within conditions (i.e., one-sample *t*-tests against zero). *P* values were corrected for multiple comparisons including all five post-hoc comparisons (i.e., 3 one sample-*t*-tests within conditions and 2 paired sample *t*-test comparing conditions).

#### Inferential statistics and effect sizes

All statistical analyses of the data were conducted in MATLAB (R2018b). Parametric tests were applied when the data met the normality assumption (*p* > 0.05 in Lilliefors test). Otherwise, appropriate non-parametric tests, including Friedman’s test and Wilcoxon signed rank test, were conducted. Mauchly’s test for sphericity was performed for all repeated-measures ANOVA models. In case of significant violations of sphericity (*p* < 0.05), Greenhouse Geisser correction was applied. The significance of effects was assessed at an alpha level of 0.05. Reported *p*-values associated with tests based on the *F*-distribution are directional, given that the *F*-distribution is not symmetrical. All conducted (non-)parametric *t*-tests were two-tailed. As measures of effect size, partial eta-squared (ηp^2^) is provided for repeated-measures ANOVA. Hedges’s *g* and *g*_1_ served as an effect size measure for paired and one-sample *t*-tests, respectively. Hedges’s *g*, and *g*1 were calculated using the MATLAB Toolbox ‘Measures of Effect Size’^60^. To correct for multiple comparisons, false discovery rate (FDR) correction was applied^61^. Corrected *p*-values are denoted as *p*_adj_.

To further facilitate the interpretation of results, we additionally report the Bayes factor (BF). While classical null hypothesis significance testing only allows conclusions on whether we can disprove the null hypothesis, the BF also allows for an assessment of whether the data favors the null hypothesis compared to an alternative hypothesis (see^62,63^ for a general introduction to Bayesian hypothesis testing). A BF greater than 3, 10, 30, and 100 provides moderate, strong, very strong, and extreme support for the alternative hypothesis^64^. Values in-between 0.33 and 3 are usually interpreted as anecdotal evidence, whereas values lower than 0.33, 0.1, 0.03, and 0.01 indicate moderate, strong, very strong, and extreme evidence in favor of the null hypothesis^62^. BFs were calculated using the MATLAB BF functions and default priors implemented by Krekelberg^64^.

## Supplementary information

Supplementary file 1

## Data Availability

The datasets generated in the course of present study are stored in the Leibniz Research Centre repository and are available from the corresponding author upon request.
