# Supplementary material for: A dual mechanism underlying retroactive shifts of auditory spatial attention: dissociating target- and distractor-related modulations of alpha lateralization
Source: Sci Rep. 2020 Aug 17;10:13860. doi: 10.1038/s41598-020-70004-2 (PMC7431585; doi:10.1038/s41598-020-70004-2)
Supplement: Supplementary file 1 [file 41598_2020_70004_MOESM1_ESM.pdf]

A dual mechanism underlying retroactive shifts of auditory spatial attention: dissociating target- and distractor-related modulations of alpha lateralization

Laura-Isabelle Klatt*, Stephan Getzmann, Alexandra Begau, & Daniel Schneider
Leibniz Research Centre for Working Environment and Human Factors

Appendix A. Supplementary Material

1. Control analyses: Lateral eye movements prior to and following retro-cue presentation

Previous related experiments from the visual domain raised concerns about alpha power asymmetries being potentially confounded by lateral shifts in gaze position[1,2]. Figure S1 shows that the frontal event-related potentials (ERP) contain a minor fixation offset towards the lateralized item in the sound array prior to retro-cue onset as well as following the retro-cue. Since gaze position has been shown to affect the perceived sound eccentricity[3], lateral saccadic eye movements may have likewise affected the electrophysiological correlates of attentional orienting towards perceived sound locations. We performed several control analyses to rule out such contamination of our data:

First of all, we calculated correlations between single-trial indices of lateral saccadic eye movements and posterior alpha power asymmetries. The ipsilateral minus contralateral difference in ERP amplitude at fronto-lateral channels F9/10 served as a measure of single-trial lateral saccadic eye movements. That is, for left-sided targets or distractors (depending on condition), ERP amplitudes at F9 minus F10 were subtracted, whereas for right-sided targets and distractors ERP amplitudes at F10 minus F9 were subtracted. Note that electrode positions F9 and F10 correspond to the most frontal channels in our EEG setup and are thus comparable to typical hEOG channel locations. Specifically, the ERP asymmetries were measured in the 200 ms preceding retro-cue onset as well as in-between 700 – 1300 ms post retro-cue onset (i.e., the same interval used for statistical analysis of alpha lateralization), accounting for lateral eye movements prior to and following retro-cue onset, respectively. Hemispheric asymmetries in the posterior alpha frequency band were analogously computed by calculating the lateralization index, as described in the methods section of the manuscript. On a single-trial level, this was done separately for right-sided and left-sided

targets/distractor. Single-trial alpha asymmetries were assessed in the same frequency range, time interval and electrode cluster used for the main analysis (i.e., 8-13 Hz, electrode cluster: PO7/8, P7/8, P5/6, PO3/4, time window: 700 – 1300 ms post retro-cue onset).

Pairwise Spearman's Rho or Pearson correlation coefficients (depending on normality properties of the data) were then calculated for each subject and each condition (i.e., target left, target right, distractor left, distractor right) as well as the two time intervals (pre- and post-retro-cue onset). After Fisher-Z transforming the correlation coefficients, one-sample *t*-tests were conducted in order to test for a statistically reliable relation between lateral eye movements and alpha lateralization in each condition. The resulting *p*-values were FDR-corrected for multiple comparisons[4] (corrected *p*-values are denoted as p_{adj}). The scatter plots in figures S2 – S5 illustrate that there was no apparent relationship between the two measures. The analysis confirmed this, revealing that in both time intervals, the single-subject correlation coefficients were not significantly different from zero, neither for target lateral nor for distractor lateral trials (all $t < .01$, $p > .9$, $p_{adj} < 2.16$, BFs < .24). Note that we did not perform this control analysis for neutral trials, since we did not observe any significant lateralization of alpha power in that condition.

In addition, since the above-mentioned correlative approach relies on the presence of null findings (i.e., a non-significant correlation between lateral saccadic eye movements and alpha lateralization), we also ran two repeated-measures analyses of covariance (ANCOVA), including the within-subject factor retro-cue type (distractor lateral vs. target lateral) and a covariate to account for the impact of lateral eye movements prior to and after the cue, respectively. That is, the first ANCOVA included the ipsilateral minus contralateral portions of the average ERP asymmetry across target-lateral and distractor-lateral conditions prior to retro-cue onset as a covariate (electrodes F9/10). This parameter did not include the neutral retro-cue condition. The second ANCOVA included the ERP asymmetry difference between distractor lateral and target lateral trials after retro-cue presentation (700 – 1300 ms post retro-cue onset) as a covariate. *P*-values were corrected for multiple comparisons across the two ANCOVAs using FDR-correction[4].

When including lateral eye movements prior to retro-cue onset as a covariate, the main effect of *retro-cue type* remained significant ($F_{(1,18)} = 12.84$, $p = .002$, $p_{adj} = .016$, $\eta p^2 = 0.42$), while there was no significant interaction of *retro-cue type* and *saccades* ($F_{(1,18)} = 3.28$, $p = .087$, $p_{adj} = .426$, $\eta p^2 = 0.15$). Analogous results were obtained when including lateral eye movements after retro-cue presentation as a covariate: The main effect of *retro-cue type* remained significant ($F_{(1,18)} = 15.79$, $p < .001$, $p_{adj} = .013$, $\eta p^2 = 0.48$), while the interaction of

retro-cue type and *saccades* was not significant ($F_{(1,18)} = 1.39$, $p = .253$, $p_{adj} = .931$, $\eta p^2 = 0.07$). Taken together, these analyses argue against any confounding influence of lateral eye movement patterns on posterior alpha lateralization.

2. Bilateral alpha power desynchronization as a measure of cognitive task demands

The line plots in Fig. 5 (a-c) clearly illustrate that there is a bilateral suppression of alpha power following retro-cue onset. Here, this desynchronization of alpha power appears to be more pronounced in distractor lateral trials than in target lateral or neutral trials. To statistically assess differences in alpha desynchronization between conditions, we performed an additional one-way repeated measures ANOVA, including the factor *retro-cue type* and bilateral, baseline-corrected alpha power as a dependent variable. Baseline-corrected ERSPs were computed, using Morlet wavelet convolution as described in the method section, but a spectral baseline was extracted for each frequency (-300 to 0 relative to pre-cue onset). Mean alpha power (8-13 Hz) was computed for each subject and the three conditions (i.e., target lateral, distractor lateral, neutral) at a posterior electrode cluster (PO7/8, P7/8, P5/6, and PO3/4) in-between 700 to 1300 ms post retro-cue onset (i.e., using the same parameters as for alpha lateralization).

The analysis revealed a main effect of *retro-cue type* ($F_{(2,38)} = 6.60$, $p = .003$, $\eta p^2 = 0.26$, $\varepsilon = .94$). Post-hoc comparisons indicated that there was a greater suppression of alpha power in distractor lateral trials compared to target lateral trials ($t_{(19)} = 3.16$, $p = .005$, $p_{adj} = .028$, $g = 0.47$, BF = 9.07) as well as compared to neutral trials ($t_{(19)} = 2.83$, $p = .011$, $p_{adj} = .030$, $g = 0.45$, BF = 4.81). In contrast, bilateral alpha suppression did not differ between target lateral and neutral trials ($t_{(19)} = 0.07$, $p = .944$, $p_{adj} = 1.731$, $g = 0.01$, $BF_{10} = 0.23$).

Such desynchronized alpha activity, resulting in low levels of alpha power (i.e., small amplitudes), has been associated with states of high excitability[5] and is commonly interpreted as a mechanism reflecting functional engagement and information processing[6]. Accordingly, the event-related desynchronization of alpha power has been associated with stimulus processing (as opposed to ‘idling’)[7], increased working memory load[8,9], and greater semantic elaboration[10]. In line with an interpretation as a signature of cognitive processing demands, distractor lateral trials in the current study presented the acoustically most challenging spatial condition, because the to-be-attended (central) sound was originally embedded by two-neighboring sounds. Thus, one may speculate that the representation generated at encoding is likely to be of lower quality than that of the lateral sound stimuli and may thus require more attentional resources to be re-focused within working memory. To follow up on this, we

performed a supplementary post-hoc analysis, comparing behavioral performance in target lateral (TL) and distractor lateral (DL) trials. In line with our interpretation, participants performed slower (M_{DL} = 874 ms, SD_{DL} = 166.93, M_{TL} = 807 ms, SD_{TL} = 151.70, $t_{(19)} = -5.88$, $p < .001$, $g = -0.41$) and less accurate (M_{DL} = 73.87 %, SD_{DL} = 10.73, M_{TL} = 81.31 %, SD_{TL} = 6.69, $z = 3.45$, $p < .001$, $U3 = 0.10$) in distractor lateral trials.

3. References

1. Schneider, D., Göddertz, A., Haase, H., Hickey, C. & Wascher, E. Hemispheric asymmetries in EEG alpha oscillations indicate active inhibition during attentional orienting within working. *Behav. Brain Res.* **359,** 38–46 (2019).
2. Rösner, M., Arnau, S., Wascher, E. & Schneider, D. Hemispheric asymmetries in posterior alpha power reflect the selection and inhibition of spatial context information in working memory. *bioRxiv* (2019). doi:10.1101/681031
3. Lewald, J. Eye-position effects in directional hearing. *Behav. Brain Res.* **87,** 35–48 (1997).
4. Benjamini, Y. & Yekutieli, D. The control of the false discovery rate in multiple testing under dependency. *Ann. Stat.* **29,** 1165–1188 (2001).
5. Haegens, S., Nácher, V., Luna, R., Romo, R. & Jensen, O. α-Oscillations in the monkey sensorimotor network influence discrimination performance by rhythmical inhibition of neuronal spiking. *Proc. Natl. Acad. Sci. U. S. A.* **108,** 19377–19382 (2011).
6. Klimesch, W., Sauseng, P. & Hanslmayr, S. EEG alpha oscillations: The inhibition-timing hypothesis. *Brain Res. Rev.* **53,** 63–88 (2007).
7. Pfurtscheller, G., Neuper, C. & Mohl, W. Event-related desynchronization (ERD) during visual processing. *Int. J. Psychophysiol.* **16,** 147.153 (1994).
8. Krause, C. M. *et al.* The effects of memory load on event-related (EEG) desynchronisation and synchronisation. *Clin. Neurophysiol.* **111,** 2071–2078 (2000).
9. Fukuda, K., Mance, I. & Vogel, E. K. A Power Modulation and Event-Related Slow Wave Provide Dissociable Correlates of Visual Working Memory. *J. Neurosci.* **35,** 14009–14016 (2015).
10. Hanslmayr, S., Spitzer, B. & Bäuml, K. H. Brain oscillations dissociate between semantic and nonsemantic encoding of episodic memories. *Cereb. Cortex* **19,** 1631–1640 (2009).

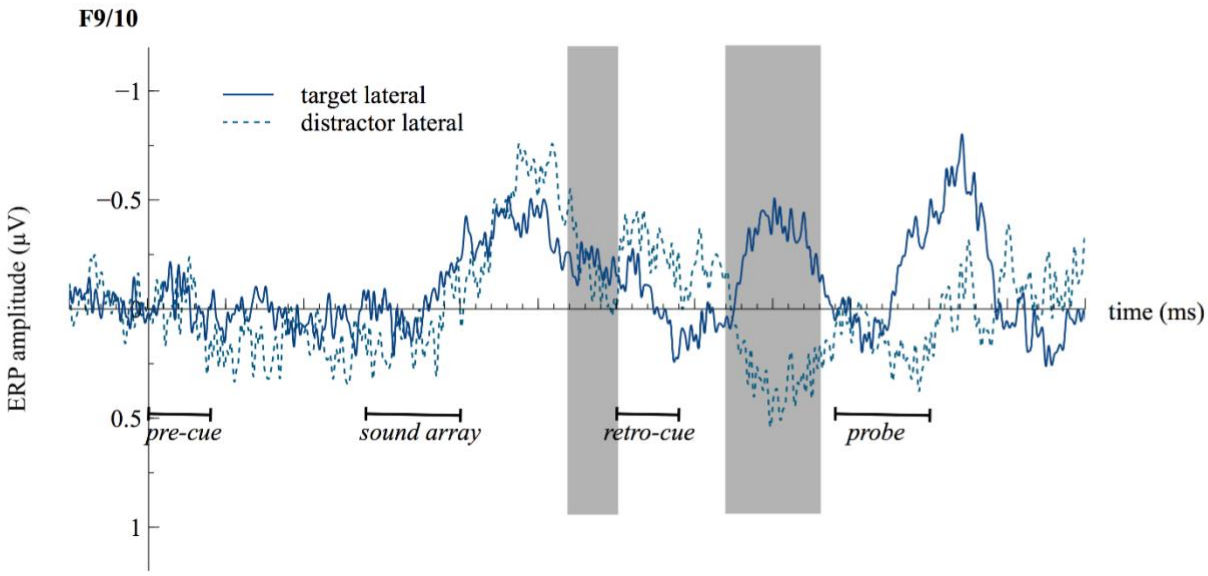

Supplementary figure S1. *Contralateral minus ipsilateral event-related potentials (ERPs) at fronto-lateral channels F9/F10. The two grey areas mark the time intervals used to extract single-trial and mean ERP amplitudes as a measure of lateral saccadic eye movements prior to and following retro-cue onset, respectively. Note that for reasons of consistency with the Alpha Lateralization Index (ALI), the analysis was performed using the ipsilateral minus contralateral amplitude differences.*

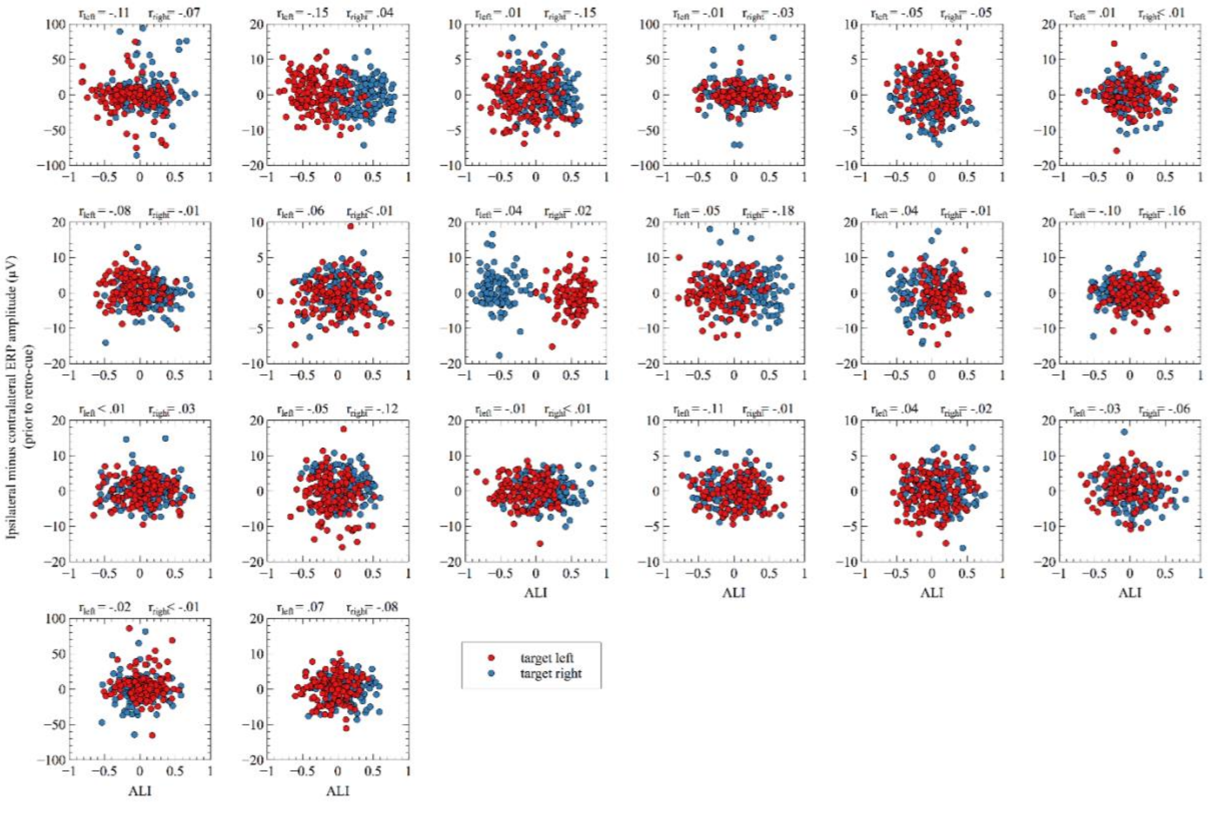

Supplementary figure S2. *Scatter plots illustrate the association between posterior alpha asymmetries and lateral saccadic eye movements prior to retro-cue onset in target lateral trials. Each plot corresponds to the data of a single subject. Single dots represent single-trial values.*

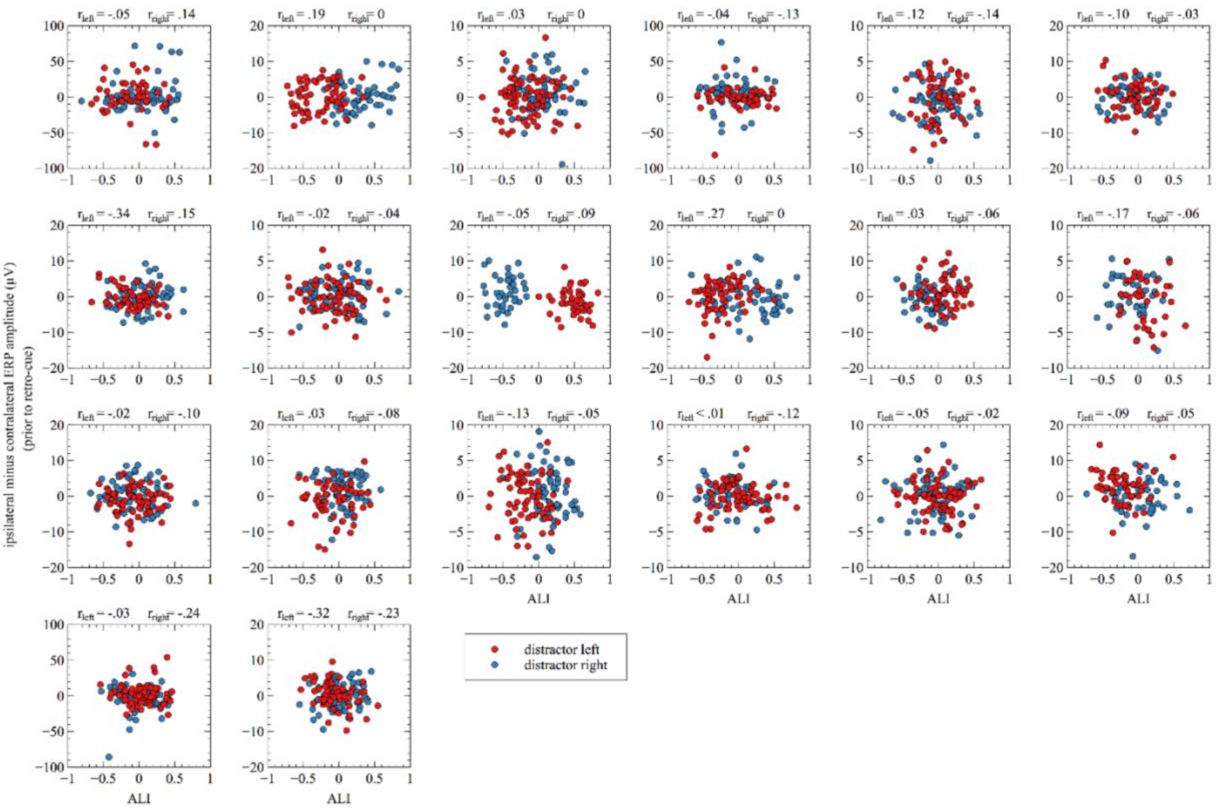

Supplementary figure S3. *Scatter plots illustrate the association between posterior alpha asymmetries and lateral saccadic eye movements prior to retro-cue onset in distractor lateral trials. Each plot corresponds to the data of a single subject. Single dots represent single-trial values.*

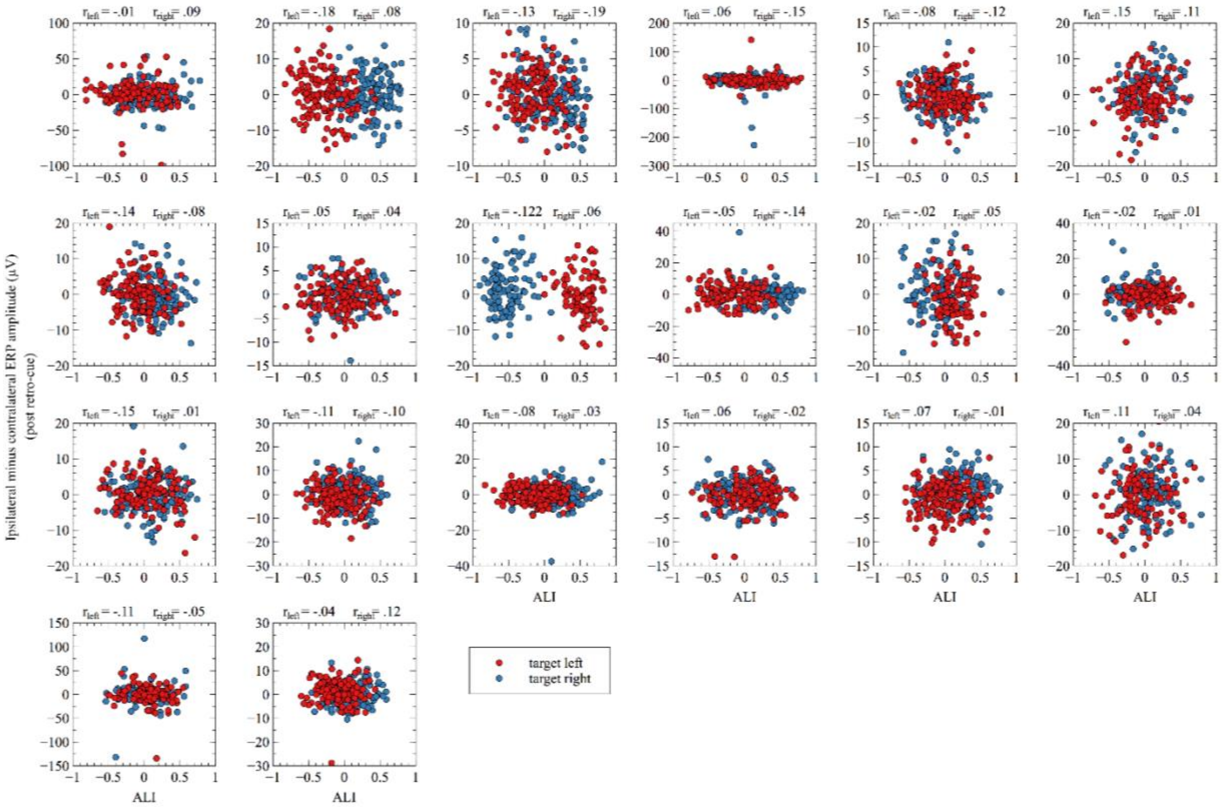

Supplementary figure S4. *Scatter plots illustrate the association between posterior alpha asymmetries and lateral saccadic eye movements following retro-cue onset in target lateral trials. Each plot corresponds to the data of a single subject. Single dots represent single-trial values.*

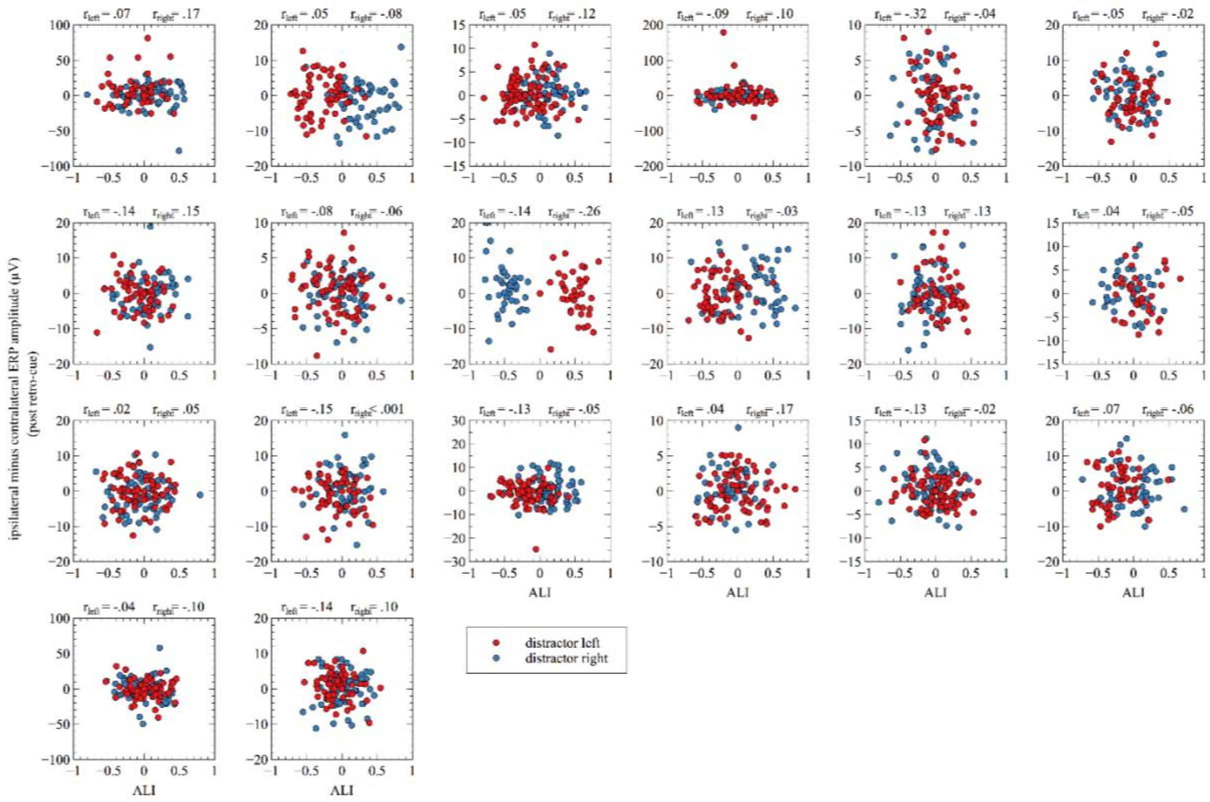

Supplementary figure S5. *Scatter plots illustrate the association between posterior alpha asymmetries and lateral saccadic eye movements following retro-cue onset in distractor lateral trials. Each plot corresponds to the data of a single subject. Single dots represent single-trial values.*
